# Changes in timber haul emissions in the context of shifting forest management and infrastructure

**DOI:** 10.1186/1750-0680-4-9

**Published:** 2009-10-29

**Authors:** Sean P Healey, Jock A Blackard, Todd A Morgan, Dan Loeffler, Greg Jones, Jon Songster, Jason P Brandt, Gretchen G Moisen, Larry T DeBlander

**Affiliations:** 1 US Forest Service, Rocky Mountain Research Station, Inventory, Monitoring and Analysis Program, 507 25thSt., Ogden, UT, 84401, USA; 2 Bureau of Business and Economic Research, School of Business Administration, University of Montana, Missoula, MT 59812, USA; 3 US Forest Service, Rocky Mountain Research Station, Human Dimensions Program, 800 E. Beckwith Ave, Missoula, MT 59801, USA

## Abstract

**Background:**

Although significant amounts of carbon may be stored in harvested wood products, the extraction of that carbon from the forest generally entails combustion of fossil fuels. The transport of timber from the forest to primary milling facilities may in particular create emissions that reduce the net sequestration value of product carbon storage. However, attempts to quantify the effects of transport on the net effects of forest management typically use relatively sparse survey data to determine transportation emission factors. We developed an approach for systematically determining transport emissions using: 1) -remotely sensed maps to estimate the spatial distribution of harvests, and 2) - industry data to determine landscape-level harvest volumes as well as the location and processing totals of individual mills. These data support spatial network analysis that can produce estimates of fossil carbon released in timber transport.

**Results:**

Transport-related emissions, evaluated as a fraction of transported wood carbon at 4 points in time on a landscape in western Montana (USA), rose from 0.5% in 1988 to 1.7% in 2004 as local mills closed and spatial patterns of harvest shifted due to decreased logging on federal lands.

**Conclusion:**

The apparent sensitivity of transport emissions to harvest and infrastructure patterns suggests that timber haul is a dynamic component of forest carbon management that bears further study both across regions and over time. The monitoring approach used here, which draws only from widely available monitoring data, could readily be adapted to provide current and historical estimates of transport emissions in a consistent way across large areas.

## Background

Significant amounts of carbon may be stored in harvested wood products. In the United States, for example, removals of atmospheric carbon due to changes in forest product pools are currently on the order of 50 Tg C per year [1,2]. However, the management activity needed to transfer ecosystem carbon to product pools usually involves the release of carbon from fossil fuels. The transport of timber from the stand to processing facilities may be a particularly important source of fossil carbon emission. Such transport can represent more than 50% of all fossil carbon emissions related to forest management [3,4]. Sonne [5] estimated that timber transport in 50-year rotation Douglas-fir forests released carbon dioxide equivalent to 4.8% of stand storage. While timber transport may represent a significant "cost" of sequestering carbon in harvested wood products, the magnitude of haul emissions will clearly vary in relation to the distance timber must travel to reach milling facilities.

To date, logistical obstacles have prevented comprehensive tracking of timber transport distances and emissions. While average road distances can be tracked by individual drivers and/or mill operators, record-keeping at the landscape level would be onerous. Existing efforts to capture haul distances and emissions (e.g. [4,6]) tend only to survey selected mills or drivers. Also, since the spatial distributions of both harvests and mills are dynamic over time, it is possible that even comprehensive estimates of haul-related emissions might quickly become out-of-date. For instance, it is broadly recognized that changes in the milling industry in the western United States over the last 2 decades have led to the closure of many local facilities and the consolidation of timber processing within a shrinking number of larger mills [7]. A possible result of this trend might be that logs must now travel farther to be processed, resulting in higher fossil carbon emissions per unit of wood product carbon.

The goals of the research described here were three-fold: 1) to demonstrate a methodology that may be used in the US and other countries having comparable monitoring resources to include *all *relevant mills in haul distance calculation, 2) to assess the degree to which transportation-related emissions reduce the net sequestration of wood product carbon in a study area in western Montana (USA), and 3) to investigate how the fossil carbon "cost" of timber transportation has changed over 2 decades in the context of shifting spatial distributions of milling facilities and changing federal forest land management.

The monitoring approach used here relied upon two well-developed, nationally available data sources: timber industry output monitoring and harvest maps produced with time series of Landsat satellite imagery. Timber output monitoring, while not originally designed to aid carbon monitoring, is a potentially rich source of information for timber transport monitoring because it captures the flow of raw materials to processing sites. In the United States, this information is collected nationally through Timber Product Output (TPO) studies carried out on behalf of the Forest Inventory and Analysis Program (FIA) of the U.S. Forest Service [8-10]. Mill censuses and logging utilization studies are conducted periodically (approximately once every 2-6 years) at the state level to account for all of the destinations of the roundwood harvested from each local area (county). The volume and species of incoming timber for each mill can be identified by county of origin.

Likely transport distances may be calculated when this record of timber destinations is combined in a geographic information system (GIS) with remotely sensed information about timber origins. The location and timing of forest harvests may be accurately mapped using historical Landsat satellite imagery. Healey et al. [11] mapped stand-clearing harvests in Oregon and Washington from 1972 (the first year of Landsat imagery availability) to 2002 in 4-year intervals with an overall accuracy of approximately 90% (kappa = 0.8). Other studies have shown similar accuracies [12-14]. Mining these two data sources may provide a more comprehensive picture of timber flows than current survey methods allow, especially over time.

Several tools have been developed in the context of carbon offset monitoring to quantify carbon sequestration related to forest harvest. Systematic efforts have been made to account for several factors, including: forest type, stand age, region, and product type [15]. However, defensible estimates for timber transport emissions are rarely available at the landscape level. More importantly, the underlying relationships between transport emissions and changes in forest management and industry infrastructure have not been explored. As the processing industry is impacted by changes in timber availability, global and local competition, the introduction of new products and processes, and changing market conditions, insight is needed into the magnitude and direction of changing transport emissions. The results of this study may shed light on underlying causes and ultimate carbon consequences of transport emission trends in the Northern Rockies region of the US. Such insights will be necessary as we try to understand the implications of forest management for the global carbon cycle.

## Results

### Estimates of Timber Volume Loss

Differences in map surfaces of predicted volume over time were used to identify the location, timing, and magnitude of harvests in Ravalli County from 1985 to 2005 in approximately 2-year intervals. These disturbance maps were subsequently used in simulations to spatially distribute TPO-recorded county-level harvest volumes for the years 1988, 1993, 1998, and 2004. Validation of the growing stock volume model used to create these surfaces was performed using independent FIA plot measurements. The model showed a root mean squared error (RMSE) of 120 cubic meters/hectare, approximately 68% of the mean observed value. This is consistent with accuracies of similar modelling efforts using the Landsat sensor [16].

**Figure 1:**
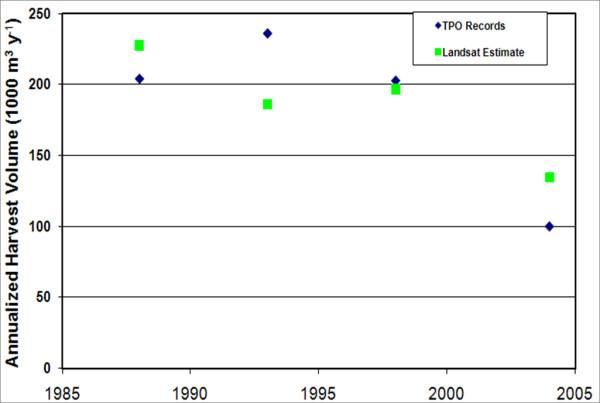
**Estimates of growing stock harvest volume using TPO records and Landsat change mapping**. Landsat-based estimates were derived through the "state model differencing" (Healey et al., 2006) approach of modelling the change in a biophysical variable such as volume over time. TPO records were available for 1988, 1993, 1998, and 2004, while Landsat intervals were: 1986 to 1988, 1993 to 1995, 1997 to 1998, and 2003 to 2005. Landsat estimates represent the total mapped harvest volume divided by the number of years in the monitoring period.

Although mapped estimates of harvest volume were used only to spatially distribute known county-level numbers, there may be some cases outside of the United States where industry monitoring data is not available. To test the compatibility of official records with estimates derived from satellite and inventory data, mapped estimates of volume removal were compared to county-level TPO harvest records. The time periods in these two monitoring efforts were not perfectly aligned. Image-based estimates were generally over 2-year periods, beginning and ending during the growing season, while the 4 dates of TPO data cover single calendar years. Nevertheless, after dividing the map-based estimates by the number of years between images, the magnitude and trend of image-based estimates corresponded well with TPO harvest records (Pearson product moment correlation coefficient 0.61; Figure 1).

### Timber Haul Distance and Carbon Emissions

The average likely road distance among the simulations performed here were: 46.3 km in 1988, 80.9 km in 1993, 82.9 km in 1998, and 214 km in 2004. Of these distances, 17.1, 13.0, 20.5, and 26.0 km respectively were over unfinished or local roads. The number of mills processing the county's timber generally went up through the study period, as did the distance of new mills from the timber-producing areas of the county. In addition to the changing orientation of mills, there was evidence that trends in the positioning of harvests patches played a small role in increasing haul distances. When 1988 harvest locations were paired via 500 simulations with 2004 mill locations (using each mill's share of the county's 2004 harvest), the average simulated haul distance was 204.1 km, which was 4.5% less than when 2004 harvests were sent to 2004 mills. Likewise, when 2004 harvests were sent to 1988 mills, average haul distances were 57.2 km (23.7% higher than when 1988 harvest locations were used). This suggests that 1988 harvest locations were closer to processing infrastructure as it existed both in 1988 and 2004. Despite this evidence that harvest positioning made a contribution to increasing haul distance, changes in haul destination (i.e. receiving mill locations) nevertheless accounted for most of the increased haul distance.

**Figure 2:**
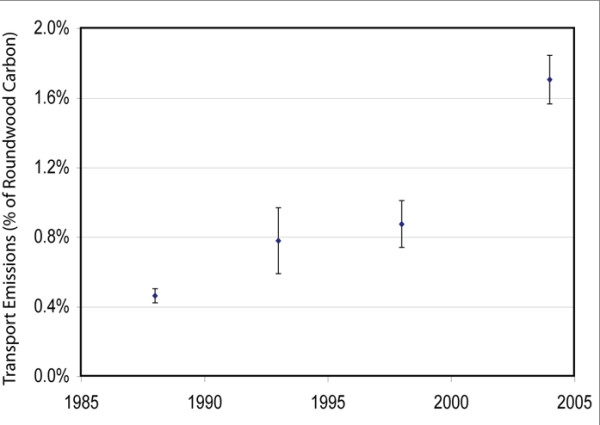
**Change in Ravalli County log transport emissions as a function of the carbon in the roundwood being transported**. Bars represent the standard deviation of 500 simulation results.

As haul distances rose throughout the period, carbon emissions as a share of the sequestered product carbon (i.e. as a fraction of the wood volume processed by mills) more than quadrupled from 1988 to 2004 (Figure 2). Bars in Figure 2 represent the standard deviation of estimates resulting from the 500 simulations for each date. Only the emission estimates for 1993 and 1998 were within each other's one-standard deviation bar.

## Discussion

Two important trends in the harvest and processing of forest products occurred between the beginning and end of the study period. According to TPO records [9,17-21], harvest volumes in federally managed national forests in the county dropped 76% between 1988 and 2004, while volumes on other lands dropped only 11%. The resulting ownership-related spatial shift in timber production was seen to increase total haul distances in this study. However, changes in the processing industry had a much larger impact on haul distance. Reductions in available timber volume contributed to the closing of many mills in the Interior West (US) region during the study period [9,22]. Although the opening of new smaller-scale log home and log furniture facilities kept the total number of firms relatively stable [9,23], the closing of higher-volume sawmills meant that the majority of logs had to be shipped farther to reach surviving mills.

Our results showed that as local mills closer to Ravalli County's timber-producing areas closed, transport emissions quadrupled in relation to the carbon sequestered in forest products. More study is needed to determine the overall carbon consequences of shifts in the processing industry, particularly in light of data showing that existing facilities have increased residue utilization, decreased residue production, and improved overall efficiency [9]. However, the changing spatial arrangement and number of operating mills had a clear impact on the transportation component of net sequestered carbon.

The GIS-based methods used in this study to identify transportation carbon emissions had several advantages over the questionnaire-type data sources commonly relied upon by product life cycle studies. First, using globally available Landsat data and nationally available industry monitoring, the system considers all mills and likely harvest sites instead of just a sample. The observed sensitivity of estimates to different configurations of timber sources and destinations suggests that any sample would have to be fairly extensive in order to be adequately representative of landscape-level dynamics. The method of emission calculation used here is straightforward, and the resulting comprehensive monitoring may not require significantly more time than conducting extensive surveys. Further, the system's simulation approach allows ready quantification of uncertainty related to the routing of the county's timber. Most importantly, this approach allows emissions monitoring that is consistent over both space and time, permitting analysis of the kinds of management and infrastructure trends discussed above.

The use of a GIS to track timber transport would allow, if needed, monitoring of a variety of transport types. While truck transport is by far the most common means of transportation in the study area and throughout much of the world [24], care should be taken to check this assumption locally. In Sweden, for example, although transportation by truck now predominates, river transport was much more common in the early 1970's [3]. Differential emission loadings could be implemented for certain routes (following river courses or rail routes, for example) in the GIS if there were evidence of significant non-truck transport. Even within a single haul, different surfaces and grades likely result in important differences in the rate of fuel consumption. Our simulations used generalized fuel efficiency rates for logging trucks in the western United States, but pending better information about the relationship between road type and fuel use, application of differential rates of combustion by road surface would increase the precision of fuel use estimates. Simulated haul distances on unfinished and local roads were reported here to demonstrate that such information may be preserved in this approach.

While the measured magnitude of fossil carbon emissions in relation to the carbon sequestered in forest products was fairly small (1.7% in 2004), realistic accounting of the effects of forest management on ecosystem carbon flux will depend upon accurate quantification of such factors. There are a number of resources designed to help landowners in the United States account for variables such as forest type and age in determining the carbon "offset" value of their managed stands [15,25-27]. Extending the methods described here to a sample of county-level transport emissions could generate systematic, regionally specific factors by which carbon offsets related to forest product sequestration could be reduced to account for haul-related emissions. Extensive historical data to support such an effort are available: TPO monitoring has occurred for decades across the country [8], and consistent maps of historical forest disturbances are available through projects such as the North American Forest Dynamics element of the North American Carbon Program [28].

Because of the availability of comprehensive, well-accepted harvest records, this study used satellite disturbance maps only to spatially distribute known harvest volumes across the landscape. However, in countries where industry monitoring is less complete or for landscapes covering political units with incompatible monitoring systems, inventory-calibrated satellite maps may be used to estimate not only the position but the magnitude of harvests [29]. In this study, TPO- and satellite-based estimates of county-level harvest volume were similar over 4 points in time (Figure 1). It should be remembered, however, that while satellite maps can depict a range of harvest intensities [30,31], they may miss harvests occurring only in the understory or that involve the salvage of timber after a natural disturbance has already removed the canopy. Therefore, projects using remote sensing as the sole source of harvest volume information (i.e. that do not use independent harvest data, as we do) should take care not to over-interpret the magnitude of emission estimates resulting from the techniques described here.

Beyond carbon accounting, the network analysis used here may have applications in decisions regarding the location of facilities designed to extract energy from forest biomass. Haul distance is a critical economic consideration in the viability of such projects, with the costs of longer hauls precluding the generation of some forms of bioenergy [32]. The network analysis presented here could easily be run using simulated harvest and facility locations to predict haul costs and identify favourable harvest-facility orientations. Only slight changes would be needed in the calculations to reflect the use of biomass container trucks instead of logging trucks, and to express distance costs in economic terms rather than as a function of carbon emissions.

## Conclusion

A full accounting of the effect of forest management on forest carbon exchange is needed if forests are to be actively integrated into plans for reducing atmospheric concentrations of greenhouse gases. This study highlighted a GIS-based method for comprehensive and consistent tracking of logging truck fossil carbon emissions. It also determined that such emissions can vary significantly in response to changes in management practices and industry infrastructure. As future efforts further characterize the carbon dynamics of complex forest landscapes, it is likely that new applications will continue to be found for existing monitoring data from satellites, forest inventory, and the timber industry.

## Methods

### Study Area and Basic Monitoring Data

Network analysis was used to determine likely haul distances in Ravalli County, Montana, during 4 years: 1988, 1993, 1998, and 2004. Ravalli County (approximately 622,000 hectares) is situated in the Northern Rocky Mountain region of the western United States. The mostly agricultural Bitterroot Valley is at the center of the county, with forested foothills and mountains toward the exterior. Forests in the county are coniferous; lodgepole pine (*Pinus contorta*) and Douglas-fir (*Psuedotsuga menziesii*) are the most common tree species by volume, with significant additional stocks of subalpine fir (*Abies lasiocarpa*), Engleman Spruce (*Picea engelmannii*), and ponderosa pine (*Pinus ponderosa*) [33]. The region has a well-established timber industry, and reliable county-level harvest data goes back at least 50 years [21].

The TPO data used in this analysis came from comprehensive censuses of mills, including those outside the county, which processed Ravalli County timber. A decision regarding the boundary of this study was made to study the transport of all materials harvested in Ravalli County to wherever they were processed. TPO data could just have easily supported the inverse of this approach: i.e. the haul distance of materials harvested anywhere and then transported to Ravalli County for processing. However, this alternative approach for generating average haul distance would have required significantly more image processing to map harvests across a broader area. In this region, mill censuses are carried out on behalf of the US Forest Service approximately every 5 years [9,17-21]. Mill census results include mill-specific volumes by species group and primary product type. While the activities of individual mills are not released to the public, mill-specific volumes and locations may be used internally in projects such as this one as long as reported analyses maintain confidentiality of firm-level proprietary information.

While TPO data defined the destinations and volumes of all timber hauls emanating from Ravalli County for the 4 years studied (1988, 1993, 1998, 2004), haul origins were described through satellite mapping and inventory data. Landsat satellite imagery was acquired from both before and after each of these dates (Table 1). All imagery was radiometrically corrected to surface reflectance using the Landscape Ecosystem Disturbance Adaptive Processing System [34].

**Table 1:** Acquisition dates for Path 41, Row 28 Landsat 5 satellite imagery used in harvest mapping.

**Year**	**Date**
1986	7 August

1988	27 July

1993	10 August

1995	31 July

1997	21 August

1998	8 August

2003	21 July

2005	11 August

This imagery was used with topographic data and forest inventory data to create a model of growing stock volume (net timber volume in the central stem) using Random Forests, a recursive, non-parametric tree-based approach [35]. Inventory data were supplied by the Forest Inventory and Analysis unit (FIA) of the US Forest Service, which maintains a consistent national network of field plots and is responsible for reporting the status and trends of the country's forest resources [36]. A total of 472 plots, taken from Ravalli and adjoining counties, were available for model building; an independent set of 337 plots was reserved for model validation. This model was developed in the R statistical environment [37] and was applied to each image in the time series using a freely available package [38].

The resultant maps of growing stock volume were subtracted from one another across time periods to produce maps showing the location and intensity of harvests in terms of estimated growing stock volume removal. Areas of volume loss greater than 17.5 cubic meters/hectare were interpreted as disturbances in each period. This threshold was determined empirically, considering the above error in the original growing stock volume model and the appearance of the resulting disturbance maps. Because many sources of error such as unusual topographic shading or rare species mixes are fairly constant for individual areas over time, changes in predicted volumes over time using even relatively weak models may be good indicators of disturbance [39]. This method of change detection is called "state model differencing" [39].

Of the disturbances identified for each of the 4 time periods, patches of harvest were visually delineated using patch spatial properties and through the elimination of likely fires using a publicly available national historical fire database [40]. The predicted harvest volume of each patch was derived from the sum of predicted volume losses (i.e. predicted pre-harvest volume minus predicted post-harvest volume) from each of the patch's constituent pixels. Overall map-based estimates of harvest volume at the county level were checked against TPO-derived records of harvest volume. Forest type, as derived from the majority value from the FIA national forest type map [41], was also attached as an attribute to each mapped harvest. The overall accuracy of this forest type map, as condensed to the 4 types used here (see Table 2) and assessed against the 89 FIA plots available for Ravalli County, was 78%. Thus, estimates of harvest location, timing, species and volume were available for subsequent network analysis, along with previously described TPO information about the volume and species of timber processed by each mill.

**Table 2:** Distribution of measured growing stock volume among species across 4 different forest types in 89 forested FIA plots in Ravalli County (measured from 2003 to 2007).

	**Species**			
	
	**Lodgepole Pine**	**Ponderosa Pine**	**Douglas-fir**	**Other**
	
				
**Forest Type**	Proportion by species			
Lodgepole pine type	0.73	0.00	0.09	0.17

Ponderosa pine type	0.01	0.87	0.10	0.02

Douglas-fir type	0.22	0.06	0.64	0.08

Other types	0.15	0.00	0.11	0.74

### Determining Carbon Emissions

Estimated emissions were based upon the assumptions that logging trucks average 1.7 km per liter of fuel [42,43], and that combustion emissions total 10.52 kg C/liter of diesel (, accessed June, 2009). Schwaiger and Zimmer [24] reported a range of payload capacities among European nations; the simulations reported here used an average capacity of 27.2 metric tonnes per logging truck, following survey results from Washington state presented by Mason et al. [43]. It was assumed that one m^3 ^of roundwood weighs 0.9 tonnes (following [24]; similar to the rate of 0.87 t/m^3^used by [3]), although variation in specific gravity certainly occurs among species. Accordingly, a factor of 0.0143 kg C/m3/km was used to identify fossil carbon emissions related to timber transport. A factor of 150% was added [3] to account for empty return trips and loading activities. Thus, the final carbon emission factor used in the simulations below was 0.0214 kg C/m^3^/km. All transport was assumed to occur via logging trucks.

### Network Analysis

Since TPO records give historical harvest volumes, the main obstacle in applying the above emission factor (0.0214 kg C/m^3^/km) is a lack of knowledge of the average haul distance for the county's timber. We used the spatial network of mapped Ravalli County harvests and known mill locations to estimate average haul distance. Simulated routings, constrained by the known timber intake of each mill, were used to explore a range of potential transportation scenarios at 4 points in time: 1988, 1993, 1998, and 2004. The major steps of this network analysis are detailed below.

#### 1. Determine likely haul distance between each mapped harvest polygon and each mill

**Figure 3:**
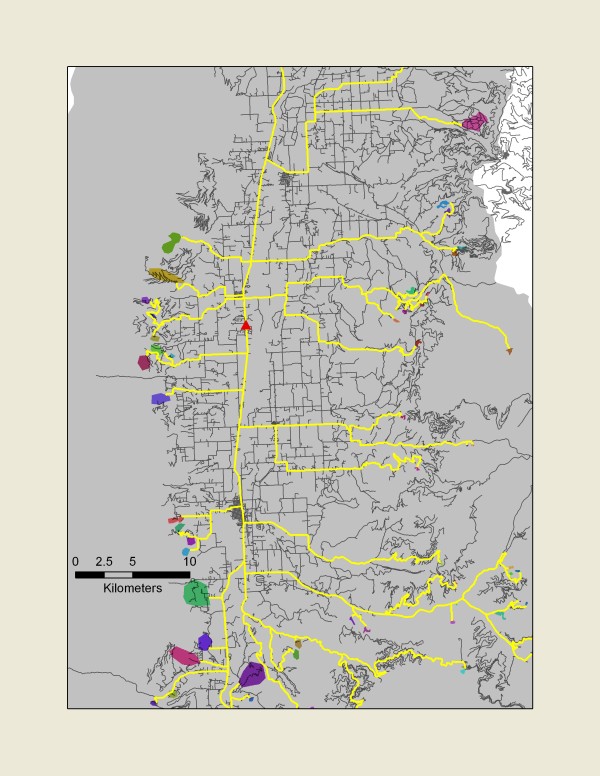
**Likely routes (yellow) identified between mapped forest harvests (colored patches) and a single mill (red triangle) in Ravalli County**. The distances of the likely routes between each mapped harvest and each mill processing Ravalli County timber were used in calculating likely haul-related fossil-carbon emissions.

Likely road distances between each pair-wise combination of mapped harvest polygons and known mill locations (hereafter called "patch-mill pairs") were determined using the "Least-Cost-Path" in the ArcGIS environment [44] (see Figure 3). Ancillary spatial data that included the region's topography (National Elevation Dataset, available from  in May, 2009) and road network were acquired. These spatial variables were weighted (see Table 3) to create a "cost" function that was applied to the region. In general, roads were given the lowest cost (with developed roads preferred over dirt roads), and in areas where road layers provided no connections (a common phenomenon in areas adjacent to harvest sites), topographic considerations became important (gentle slopes preferred over steep). The algorithm identified the route minimizing this cost function. For each patch-mill pair, both the total likely road distance and the distance on unfinished roads (local or rural roads, vehicular trails, areas with no recorded roads) were written to matrices that could be referenced in later stages.

**Table 3:** Factor and field weights used in the cost-spread function that produced likely haul routes and distances.

**Factor**	**% Influence**	**Field**	**Scale Value**
Road Type	80	Interstate highway	1

		US highways	3

		State & County highways	5

		Local or rural road, city street	7

		Vehicular trail	12

		Miscellaneous (traffic circle, access ramp, etc.)	20

		Private drives or foot trails	30

		No coded road	400

Slope	20	0-10%	1

		10-20%	5

		20-30%	10

		30-40%	15

		40-50%	20

		50-60%	30

		60-70%	40

		70-80%	50

		80+%	60

Scale values may be thought of as factors used to determine the path of least resistance from each harvest to each mill.

#### 2. Distribute TPO-recorded harvest volume to mapped patches

Satellite-based harvest maps were used to determine the spatial distribution of TPO-recorded county-level harvest volumes. Because sorting and separation of different species is common at the harvest site, 4 different species bins- lodgepole pine, ponderosa pine, Douglas-fir, and a group containing less common species - were "routed" independently within the simulations. As described earlier, a modelled volume loss could be attributed to each patch. That patch-level volume was first distributed among the 4 species bins. Distribution by species used each patch's majority value according to a widely used forest type map [41]. This forest type call was combined with a survey-based county-level breakdown of the distribution of volume among species within each forest type [33] (see Table 2) to determine how much of the mapped removal was attributable to each species.

Modelled harvest volumes for each species from each patch were then scaled to TPO-derived county-level numbers. Specifically, each harvest patch's fraction of all mapped harvest volume within the county for a given species was multiplied by the actual TPO-based county-level harvest volume for that species. In this way, the overall amount of timber being moved in the simulation matched TPO records and was distributed among mapped harvest patches in proportion to those patches' estimated harvest volume. This also had the effect of reconciling the 2-year mapping intervals (starting and ending during mid-year growing seasons) with the calendar-year time steps of the TPO data and this study.

#### 3. For each simulation, generate a list of mill-patch routes

The preceding steps assigned a harvest volume to mapped harvest patches across the landscape and also identified the location and capacity of mills processing that harvest volume. Still unknown, however, was the routing of timber from each origin (patch) to each destination (mill). 500 simulations involving different pairings of patches and mills were used to develop an understanding of the trend and variability of possible haul distances. Proximity was not incorporated as a factor in the choice of routings because many of the harvests were relatively close to one another and because of professional judgment that differences of a few kilometers are not typically the basis for the choice of a processing facility.

In each simulation, the percentage of patches "sending" their estimated harvest volume to a particular mill was approximately equivalent to that mill's proportion of the county's total harvest (according to TPO records). For every species bin in each patch, a random number between 0 and 1.0 was generated in relation to each mill. Patch-mill pairs were flagged and later included in the simulation if the random number was less than the mill's TPO-derived share of the county's harvest volume for that species. This process was carried out independently for each harvest site and mill, so the volume from a given harvest was not forced to go to exactly one mill. In aggregate, however, harvest volume from each patch was sent to an average of one mill, and the known capacities of each mill were realistically represented. If a mill processed 30% of Ravalli County's wood products, approximately 30% of the harvests in each realization would be randomly flagged as points of origin for that mill.

#### 4. For each chosen route, apply emission formula, sum emissions within the simulation, and combine results over all simulations

The list of selected patch-mill pairs generated in the previous step could be related to both the matrix of "least-cost" road distances between each patch and each mill, and to the estimated harvest volume for each patch (divided among 4 species bins and scaled to TPO county-level harvest records). As a result, the transportation emissions associated with the routings selected in each simulation could be calculated using the emission factor calculated above (0.0214 kg C/m^3^/km). These emissions were summed over all selected mill-patch pairs within the simulation to produce an estimate of total transport emissions. The carbon content of the total county-level volume of transported timber was estimated using a factor of 13.42 kg C/m^3 ^of roundwood, the value given by Skog and Nicholson [45] as the carbon content of "Northern Rocky Mountain softwoods." Total emissions for each simulation were divided by the estimated carbon content of the transported wood to produce a ratio of fossil carbon emitted to product carbon transported.

The average of this ratio over all simulations was taken as an estimate of the transportation emission rate, with the standard deviation providing a measure of uncertainty.

## Competing interests

The authors declare that they have no competing interests.

## Authors' contributions

SPH performed remote sensing, simulations and was primary author; JAB performed cost-spread analysis: TAM, JS, and JBP analyzed historical TPO data and contributed to Discussion, DL and GJ contributed to simulation design, and GGM and LTD contributed to volume modelling and interpretation of FIA data. All authors read and approved the final manuscript.
